# Direct evidence that the N-terminal extensions of the TAP complex act as autonomous interaction scaffolds for the assembly of the MHC I peptide-loading complex

**DOI:** 10.1007/s00018-012-1005-6

**Published:** 2012-05-27

**Authors:** Sabine Hulpke, Maiko Tomioka, Elisabeth Kremmer, Kazumitsu Ueda, Rupert Abele, Robert Tampé

**Affiliations:** 1 Biocenter, Institute of Biochemistry and Cluster of Excellence Frankfurt (CEF) - Macromolecular Complexes, Goethe-University Frankfurt, Max-von-Laue-Str. 9, 60438 Frankfurt am Main, Germany; 2 Laboratory of Cellular Biochemistry, Division of Applied Life Sciences, Graduate School of Agriculture and Institute for Integrated Cell-Material Sciences (iCeMS), Kyoto University, Kyoto, 606-8502 Japan; 3 Helmholtz-Center Munich, German Research Center for Environmental Health, Institute of Molecular Immunology, Marchioninistr. 25, 81377 Munich, Germany

**Keywords:** ABC transporter, Antigen processing, Membrane protein interaction, Macromolecular membrane complex, Tapasin

## Abstract

The loading of antigenic peptides onto major histocompatibility complex class I (MHC I) molecules is an essential step in the adaptive immune response against virally or malignantly transformed cells. The ER-resident peptide-loading complex (PLC) consists of the transporter associated with antigen processing (TAP1 and TAP2), assembled with the auxiliary factors tapasin and MHC I. Here, we demonstrated that the N-terminal extension of each TAP subunit represents an autonomous domain, named TMD_0_, which is correctly targeted to and inserted into the ER membrane. In the absence of coreTAP, each TMD_0_ recruits tapasin in a 1:1 stoichiometry. Although the TMD_0_s lack known ER retention/retrieval signals, they are localized to the ER membrane even in tapasin-deficient cells. We conclude that the TMD_0_s of TAP form autonomous interaction hubs linking antigen translocation into the ER with peptide loading onto MHC I, hence ensuring a major function in the integrity of the antigen-processing machinery.

## Introduction

The adaptive immune system of jawed vertebrates is responsible for detection and elimination of virus-infected or malignantly transformed cells, thus playing an essential role in survival. Information about the cellular proteome is presented to cytotoxic T cells on the cell surface in the form of complexes of MHC I molecules with antigenic peptides derived from intracellular proteins [1–3]. A large portion of the cellular proteome is degraded by the proteasome. A fraction of these antigenic peptides is transported into the lumen of the endoplasmic reticulum (ER) by the transporter associated with antigen processing (TAP), a heterodimeric ABC complex composed of TAP1 (ABCB2) and TAP2 (ABCB3). The loading of peptides onto MHC I takes place within the peptide-loading complex (PLC), a multisubunit machinery consisting of TAP1/2, MHC I heavy chain/β_2_-microglobulin, the chaperone calreticulin, the oxidoreductase ERp57, and tapasin (Tsn). The latter is an essential adapter molecule within the PLC, as it bridges the peptide donor (TAP) with the peptide acceptor (MHC I) [4, 5]. For a large number of MHC I alleles, tapasin is required for loading of high-affinity peptides onto MHC I, a process known as peptide editing [6, 7]. After loading their peptide cargo, MHC I complexes travel via the secretory pathway to the cell surface. Large efforts have been made to understand the assembly, organization, and function of the PLC. For most ER-lumenal parts, structural information is available [8–12]. However, TAP cannot yet be crystallized, and only a homology model of the coreTAP complex is available based on the X-ray structures of bacterial ABC transporter Sav1866 and the NBD1 of TAP1 [13–15].

Each coreTAP subunit consists of six transmembrane helices plus the nucleotide-binding domain. CoreTAP shares significant homology with other ABC transporters and is necessary and sufficient for peptide binding and transport [16]. In contrast, the unique N-terminal domain with four putative transmembrane-spanning segments, called TMD_0_, shares no homology to any other known protein. Deletion of the first transmembrane-spanning segment of TAP destroys its ability to interact with tapasin [17]. However, there is no structural information available for the TMD_0_ of TAP1 and TAP2. Despite their critical role in PLC assembly, the TMD_0_s constitute the least understood part of this macromolecular machinery. TMD_0_ of TAP2 (hereafter named TMD_0_^TAP2^) is about 30 amino acids shorter than that of TAP1 (TMD_0_^TAP1^). Although their sequences markedly differ, both TMD_0_s seem to fulfill similar functions in tapasin binding [16], in spite of data indicating a functional asymmetry of greater significance of the rat TMD_0_^TAP2^ in PLC function [18]. In this study, the functionality and structural integrity of the TMD_0_s of both TAP subunits were addressed with regard to their subcellular localization, binding, and stoichiometry of tapasin.

## Materials and methods

### Cloning and constructs

TMD_0_^TAP1^ and TMD_0_^TAP2^ were cloned into pcDNA3.1(+) (Invitrogen, Darmstadt, Germany) via XhoI/EcoRI and KpnI/NotI, respectively. To generate TMD_0_^TAP1^ (aa 1–164 of TAP1) with a C-terminal myc-tag and TMD_0_^TAP2^ (aa 1–127 of TAP2) with a C-terminal HA-tag, the following primer pairs were used: 5′-GTCGACGAATTCATGGCTAGCTCTAGGTG-3′ and 5′-GTCGACCTCGAGTCACAGATCCTCTTCTGAGATGAGTTTTTGTTCGGATCCGCCGGGCACCCAG-3′ for TMD_0_^TAP1^, 5′-GTCGACGGTACCAGATCTACCATGCGGCTCCCTGACCTG-3′ and 5′-GTCGACGCGGCCGCTCAAGCGTAGTCTGGGACGTCGTATGGGTAGGATCCCTTCTCCTGGGCTCC-3′ for TMD_0_^TAP2^. CoreTAP1 (amino acids 165–748, containing an N-terminal methionine) was amplified with primer pairs 5′-CGATTACTCGAGATGGGTCAGGGCGGCTC-3′and 5′-CGATTAGAATTCCCTTCTGGAGCATCTGC-3′ and cloned into pEGFP-N3 (BD Biosciences, Franklin Lakes, NJ, USA) via XhoI and EcoRI sites. CoreTAP2-mCerulean (amino acids 125-716 plus N-terminal methionine) containing a C-terminal StrepII-tag was amplified with the primer pair 5′-CATGCTTAAGATGGCCCAGGAGAAGGAGCAGGACC-3′and 5′-CCGCTCGAGTCACTTCTCGAATTGTGGGTGAGACCAAGC-3′, then cloned into pcDNA3.1(+) via AflII and XhoI. Tsn-TMD_0_ constructs were amplified via PCR with the following primers: 5′-AGATCTATGAAGTCCCTGTCTCTGCTCCTCG -3′ as forward primer for both constructs, 5′-ATCGCGGCCGCTCACAGATCCTCTTCTGAGATGAGTTTTTGTTCACCTCCAGGCACCCAAAGACTACC-3′ for Tsn-TMD_0_^TAP1^ containing a C-terminal myc-tag and 5′-GTCGACGCGGCCGCTCAAGCGTAGTCTGGGACGTCGTATGGGTAGGATCCTTTTTCTTGGGCACCTGGTGGAC-3′ for Tsn-TMD_0_^TAP2^ with a C-terminal HA-tag. A 34 amino acid long flexible glycine-serine linker was inserted between the C-terminus of tapasin and the N-terminus of the TMD_0_. Both constructs were cloned into pcDNA3.1(+) via BamHI/NotI. For the TAP1/TAP2 coexpression plasmid, TAP1 was cloned into the MCS2 of pVitro2-neo-mcs (Invivogen, San Diego, CA, USA) via BglII and NheI, TAP2 was cloned into MCS1 via BamHI and SalI.

### Cell lines and transfection

HeLa cells were cultured in DMEM (PAA Laboratories, Pasching, Austria) supplemented with 10 % fetal calf serum (FCS; Biochrom, Berlin, Germany). M553, a human tapasin-deficient melanoma cell line [19, 20], was maintained in RPMI 1640 with 10 % FCS. Transfection of M553 was performed using XtremeGene HP (Roche, Grenzach-Wyhlen, Germany) according to the manufacturer’s instructions. HeLa cells were transfected with 18 mM branched polyethyleneimine (PEI) with a DNA-to-PEI ratio of one to three. After 24–48 h, cells were harvested and used for the indicated experiments.

### Antibodies

For immunofluorescence experiments, the following antibodies were used: mouse anti-myc 4A6 (Millipore, Billerica, MA, USA), mouse anti-HA HA-7 (Abcam, Cambridge, UK), mouse anti-TAP1 148.3 [21], and mouse anti-StrepII (IBA BioTAGnology, Göttingen, Germany). As organelle makers, rabbit anti-Calreticulin (polyclonal IgG fraction; Sigma-Aldrich, Steinheim, Germany), anti-ERGIC-53 (Sigma-Aldrich), and anti-GM130 (*Golgi*; EP892Y; Abcam) were used. Detection of the primary antibodies was done with donkey anti-mouse-Alexa488 (Invitrogen), goat anti-rabbit-Cy3 (Dianova, Hamburg, Germany), and, for simultaneous detection of GFP and StrepII together with organelle marker, goat anti-mouse-Alexa633 (Invitrogen). For immunoblotting, anti-myc 4A6, anti-HA HA-7, anti-SRP54 (BD Bioscience), anti-MHC I hc HC10 [22] and mAb 7F6, raised against amino acids _21_GPAVIECWFVEDASGKG_35_ of human tapasin, were used.

### Membrane preparation and carbonate extraction

Membranes were prepared from 5 × 10^6^ transiently transfected HeLa cells. The cell pellet was mixed with 50 mM ice-cold Tris buffer (pH 7.3) containing 250 mM sucrose and protease inhibitor mix (Serva, Heidelberg, Germany). Cells were pulped with a glass homogenizer. Post-nuclear supernatants were collected by centrifugation for 10 min at 700*g* and membranes were sedimented by centrifugation at 100,000*g* for 30 min at 4 °C. Membranes were resuspended in 0.1 M Na_2_CO_3_ buffer pH 11.5 and incubated on ice for 15 min, followed by centrifugation at 100,000*g* for 20 min at 4 °C. The supernatant was collected. The remaining pellet was resuspended in carbonate buffer pH 11.5, adjusted to 1.6 M sucrose and overlaid with 1.25 M and 0.25 M sucrose in the same buffer. Centrifugation was performed for 90 min at 100,000*g* and the floating membranes were collected. Proteins were precipitated by chloroform–methanol and subsequently analyzed by SDS-PAGE (15 %).

### Immunofluorescence and image processing

Transiently transfected HeLa cells grown on cover slips were fixed 30 min with 4 % formaldehyde in PBS at room temperature, quenched with 50 mM glycine for 10 min, and permeabilized with 0.1 % Triton X-100 for 20 min. After blocking with 5 % bovine serum albumin for 30 min, cells were stained with the primary antibodies followed by secondary antibodies for 1 h at room temperature. Nuclei were visualized by DAPI staining (Dianova). Preparations were mounted in 10 % (w/v) Mowiol (Calbiochem/Merck, Nottingham, UK). Samples were analyzed with a confocal laser-scanning microscope (LSM 510; Zeiss, Germany) equipped with a Plan-Apochromat 63x/1.4 Oil DIC objective. Deconvolution of the images was performed using non-blind 2D deconvolution of AutoQuantX2 (MediaCybernetics, Bethesda, MD, USA). Non-transfected cells were removed from the picture prior to determination of Pearson’s coefficients, and a threshold was manually set to remove background. For colocalization analysis, the JACoP plug-in [23] for ImageJ was used to calculate Pearson’s coefficients out of 8–10 individual cells per construct and organelle marker. For coreTAP1 and coreTAP2 co-expression, Pearson’s coefficients were calculated for coreTAP1-GFP and the organelle marker.

### Coimmunoprecipitation

For coimmunoprecipitation, sheep anti-mouse Dynabeads (Invitrogen) were loaded with mouse anti-myc 4A6 (TMD_0_^TAP1^ and Tsn-TMD_0_^TAP1^) or mouse anti-HA HA-7 (TMD_0_^TAP2^ and Tsn-TMD_0_^TAP2^) for 2 h at 4 °C. Control precipitations were performed using a non-specific isotype-matched mouse antibody. After coating, the beads were washed three times with 1 ml IP buffer containing 20 mM Tris/HCl, pH 7.4, 0.1 % BSA, 150 mM NaCl, and 5 mM MgCl_2_. For each sample, 2.5 × 10^6^ HeLa cells were plated in a 14.5-cm dish the day before the transfection. Then, 24 h (Tsn-TMD_0_s) or 48 h (TMD_0_s) after transfection, the cells were harvested and solubilized in 1 ml buffer containing 20 mM Tris/HCl, pH 7.4, 150 mM NaCl, 5 mM MgCl_2_, 1 % digitonin, and 1 % protease inhibitor mix (Serva) for 1 h at 4 °C. Solubilized cells were centrifuged for 30 min at 100,000*g* at 4 °C. After pre-clearing, the supernatant was incubated with pre-coated magnetic beads for 1 h at 4 °C. After washing, beads were isolated by a magnet and bound protein was eluted with 30 μl SDS sample buffer for 10 min at 65 °C. Eluates were analyzed by SDS-PAGE and subsequent immunoblotting.

### Protease K digestion

Membranes were prepared as described above. After centrifugation at 100,000*g*, membranes were resuspended in ice-cold PBS containing 5 mM CaCl_2_. Proteinase K (0.6 units, Sigma Aldrich) were added in the absence or presence of 0.1 % Triton-X 100, and incubated on ice for 30 min. Digestion was stopped by the addition of PMSF (5 mM final), followed by incubation at 95 °C for 10 min in SDS sample buffer. Samples were separated by SDS-PAGE (10 %) and subsequent immunoblotting with the indicated antibodies.

## Results

### Expression and membrane insertion of the isolated TMD_0_s

**Fig. 1:**
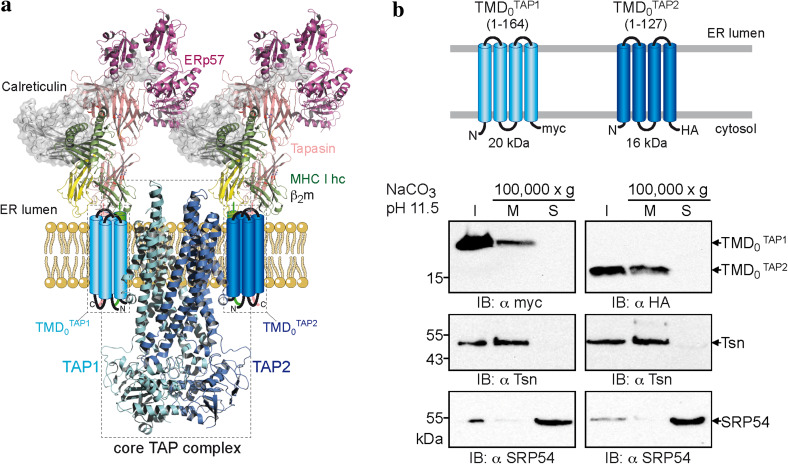
Unique N-terminal domains (TMD_0_s) of TAP1 and TAP2 represent an autonomous membrane protein fold. **a** Structural model of the TAP complex [3, 11, 50]. CoreTAP is shown as a homology model based on the structure of Sav1866 [13, 15]. The extra N-terminal domain TMD_0_ of each TAP subunit, illustrated as a four-helix bundle, recruits the tapasin-ERp57 conjugate via transmembrane domain interaction. **b** TMD_0_^TAP1^ and TMD_0_^TAP2^ are integral membrane domains. Membranes were prepared from transiently transfected HeLa cells and alkaline extraction was performed. *I* an aliquot (1/10) of the membranes before alkaline extraction to confirm expression, *M* and *S* membrane fraction and supernatant after treatment with carbonate buffer pH 11.5, respectively. Immunoblots were developed against TMD_0_^TAP1/2^, tapasin as integral type I membrane protein, and SRP54 as peripheral membrane protein

The coreTAP subunits can be expressed without affecting membrane targeting, insertion, folding, dimerization, and transport activity [16]. However, it remains open whether the unique and extra TMD_0_ represents an autonomous domain with the ability to fold independently of coreTAP and other components of the peptide-loading complex. We thus divided human TAP1 and TAP2 into TMD_0_ and coreTAP to further study their functions (Fig. 1a). The boundary for dissection of the domains lies within the second cytoplasmic loop, resulting in TMD_0_^TAP1^ (aa 1–164) and TMD_0_^TAP2^ (aa 1–127). For immunodetection, the TMD_0_s of TAP1 and TAP2 were tagged with a C-terminal myc- and HA-epitope, respectively (Fig. 1b). Strikingly, both TMD_0_s were expressed at high levels in transiently transfected HeLa cells. TMD_0_^TAP1^ as well as TMD_0_^TAP2^ were found to be in the membrane fraction (20 and 16 kDa, respectively) together with the integral type I membrane protein tapasin and resistant to alkaline extraction, whereas the peripheral membrane protein, the signal recognition particle SRP54, is in the supernatant (Fig. 1b). These results demonstrate that the TMD_0_ of each TAP subunit is targeted and inserted as integral membrane proteins.

### Subcellular localization of the TAP domains

**Fig. 2:**
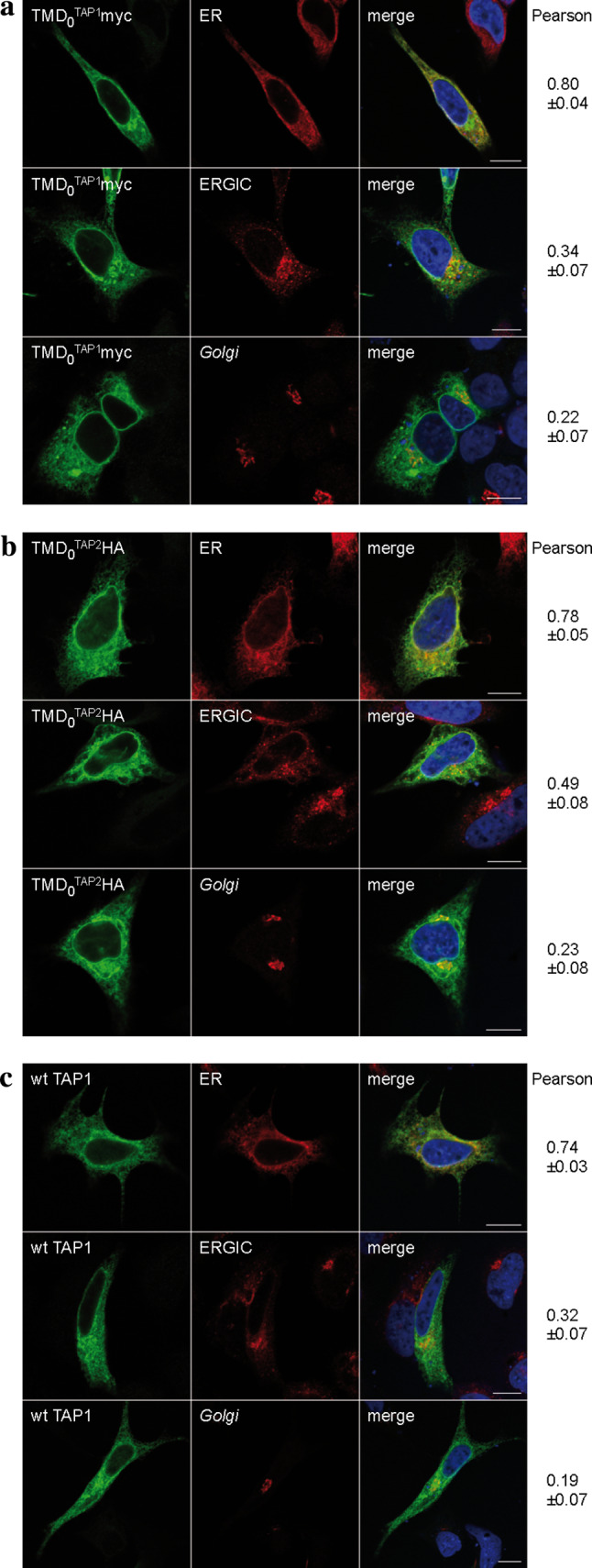
Subcellular localization of TMD_0_^TAP1/2^. Indirect immunofluorescence on transiently transfected HeLa cells (TMD_0_s or wt TAP1/2 as a control) was performed using TMD_0_^TAP1^, TMD_0_^TAP2^ and TAP1 specific (myc, HA and 148.3, respectively, *green*) as well as organelle specific antibodies [*ER* anti-calreticulin; ER-*Golg*i intermediate compartment (*ERGIC*): ERGIC-53; *Golgi* GM-130; *red*]. **a** TMD_0_^TAP1^, **b** TMD_0_^TAP2^, **c** wt TAP. Images were taken using confocal laser scanning microscopy. Pearson coefficients were calculated from 8–10 individual images. *Scale bar* 10 μm

The ER is the major compartment for MHC I peptide loading, although TAP activity was also found in post-ER compartments [24]. We therefore investigated the subcellular localization of the N-terminal domains. HeLa cells were transiently transfected with each TMD_0_ or wt TAP and analyzed by immunofluorescence microscopy (Fig. 2). As expected, wt TAP was found to be mostly in the ER. Both TMD_0_^TAP1^ and TMD_0_^TAP2^ co-localized to a large extent with the ER. Minor fractions of both TMD_0_s were found in the ERGIC. Interestingly, the overlap with the ERGIC was more pronounced for TMD_0_^TAP2^ than for TMD_0_^TAP1^. The colocalization with the *Golgi* marker GM130 was only weak, demonstrating that isolated TMD_0_s are efficiently retained in the early secretory pathway.

**Fig. 3:**
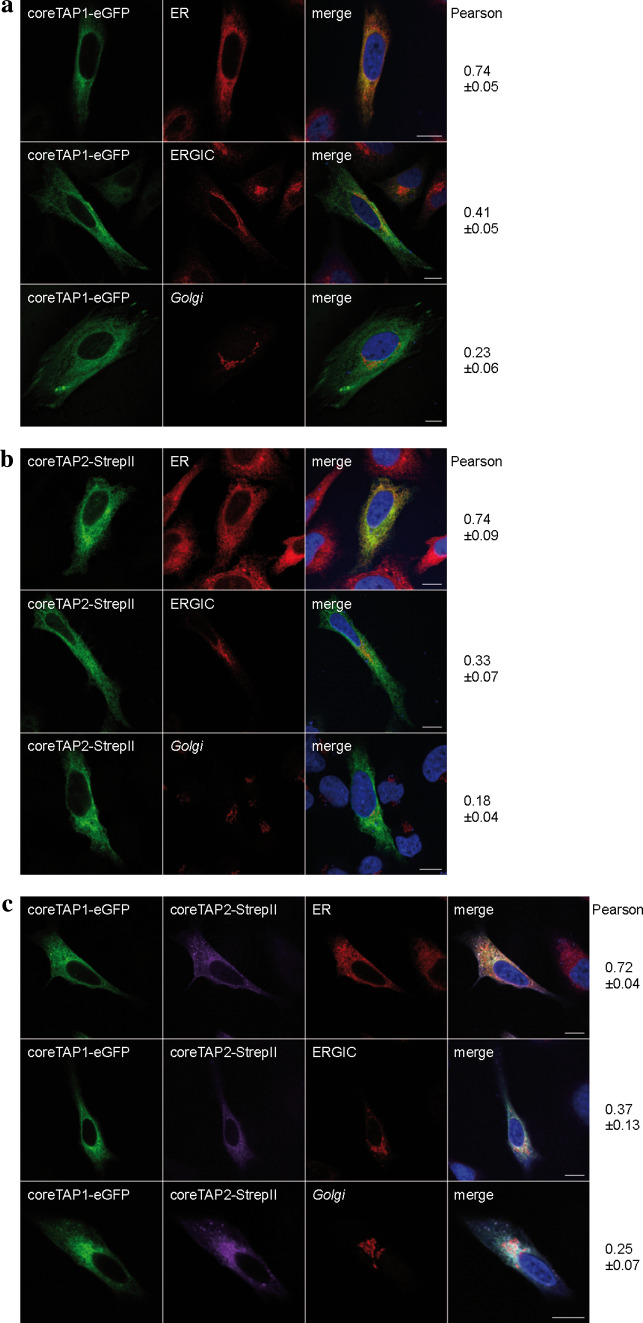
Subcellular localization of coreTAP. HeLa cells were transiently transfected with coreTAP1-eGFP (**a**), coreTAP2-mCerulean (**b**), or a combination of both coreTAP constructs (**c**), and analyzed by confocal laser scanning microscopy. CoreTAP2-mCerulean was indirectly visualized via its C-terminal StrepII-tag with a StrepII antibody. Organelles were stained with antibodies against marker proteins [*ER* anti-calreticulin; ER-*Golg*i intermediate compartment (*ERGIC*): anti-ERGIC-53; *Golgi*: anti-GM130]. Pearson coefficients were calculated from 8 to 10 individual images. *Scale bar* 10 μm

We next investigated the localization of coreTAP. Previous studies demonstrated the peptide transport activity of the coreTAP complex into ER-derived microsomes from *Sf*9 cells using a glycosylation dependent assay [16]. Due to differences in the post-translational modifications between the insect cell and mammalian cell system, this evidence for ER retention of the coreTAP complex might not necessarily be transferrable to human cells. To clarify this point, coreTAP1 and coreTAP2 were fused C-terminally to eGFP and mCerulean, respectively. Confocal laser scanning microscopy directly visualized coreTAP1-eGFP, whereas coreTAP2 was indirectly detected via its C-terminal Strep-tag, due to its low expression level and the relatively weak fluorescence of the mCerulean. Similar to the TMD_0_s, coreTAP1 and coreTAP2 localized to the ER when expressed individually in HeLa cells (Fig. 3a, b). We reasoned that this ER localization might be caused by the formation of heterodimers with endogenous TAP. To examine if heterodimers of both coreTAP subunits were also restricted to the ER, we coexpressed coreTAP1 and coreTAP2 (Fig. 3c). The Pearson’s coefficients for the tested compartments were similar to those of overexpressed wt TAP (Fig. 2c), indicating that the coreTAP shows the same subcellular distribution as wt TAP.

### TMD_0_s of TAP1 and TAP2 form autonomous platforms for tapasin recruitment

**Fig. 4:**
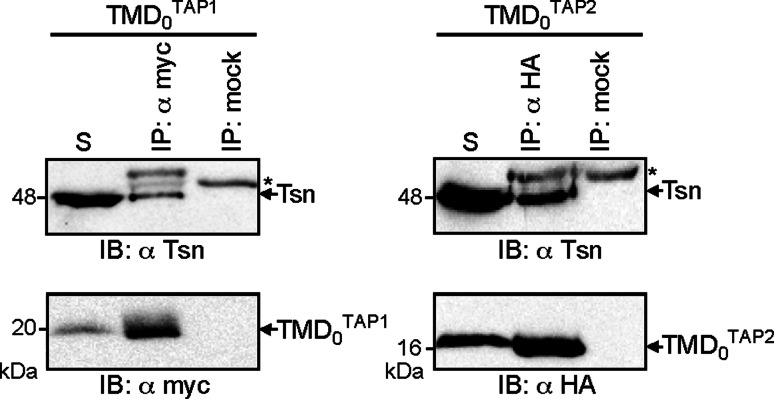
Recruitment of tapasin by TMD_0_^TAP1/2^. TMD_0_^TAP1^ or TMD_0_^TAP2^ were transiently expressed in HeLa cells and subjected to coimmunoprecipitation using myc or HA antibodies. As mock control, an isotype antibody was used. For both TMD_0_s, co-precipitation of tapasin was observed. *Asterisks*: immunoglobulin heavy chain. The solubilizate (*S*) represents 1/20 aliquot of the precipitate

It has been previously demonstrated that the coreTAP complex does not interact with tapasin [16, 17, 25]. Thus, the tapasin-binding site could be formed at the interface of TMD_0_ and coreTAP, or by the TMD_0_ alone. To distinguish between these possibilities, coimmunoprecipitation experiments were performed with the TMD_0_s of both TAP subunits and endogenous tapasin in HeLa cells. TMD_0_^TAP1^ and TMD_0_^TAP2^ were precipitated using anti-myc or anti-HA antibody, respectively (Fig. 4). Importantly, each TMD_0_ interacts with tapasin, confirming that the TMD_0_s are correctly folded and form an independent interaction domain for tapasin.

### TMD_0_s of TAP1 and TAP2 localize to the ER independently of tapasin

**Fig. 5:**
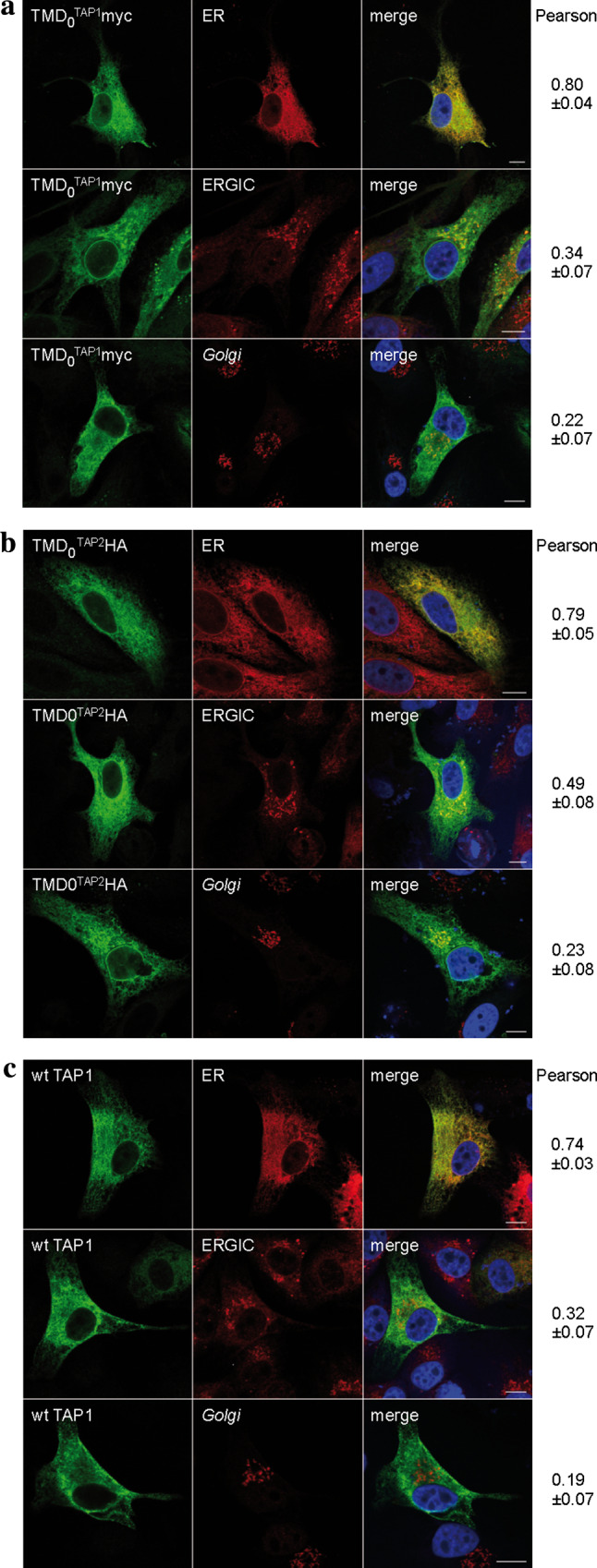
Subcellular localization of TMD_0_^TAP1^ and TMD_0_^TAP2^ in tapasin deficient cells. TMD_0_^TAP1^ (**a**), TMD_0_^TAP2^ (**b**), and wt TAP1/2 (**c**) were transiently expressed in the tapasin deficient cell line M553, stained with the antibodies as indicated in Fig. 2, and analyzed via indirect immunofluorescence. Organelles were stained with antibodies against marker proteins [*ER* anti-calreticulin; ER-*Golg*i intermediate compartment (*ERGIC*): anti-ERGIC-53; *Golgi*: anti-GM130]. Pearson coefficients were calculated from 8 to 10 individual images. *Scale bar* 10 μm

TMD_0_^TAP1^ and TMD_0_^TAP2^ lack any known ER retention signal but interact with tapasin, which harbors a di-lysine ER retrieval signal [26]. We therefore asked whether binding to tapasin retains the TMD_0_ in the ER. To address this, we expressed wt TAP or the TMD_0_s in the tapasin-deficient cell line M553 [19, 20] and analyzed their subcellular distribution by immunostaining (Fig. 5). Similar to HeLa cells, wt TAP co-localized with the ER marker and only a minimal fraction was found in the ERGIC and *Golgi*. The same distribution was observed for TMD_0_^TAP1^ and TMD_0_^TAP2^, demonstrating that tapasin interaction is expendable for correct localization. Again, the fraction of TMD_0_^TAP2^ that was found in the ERGIC was enhanced compared to that of TMD_0_^TAP1^.

### Each TMD_0_ provides one single binding site for tapasin

**Fig. 6:**
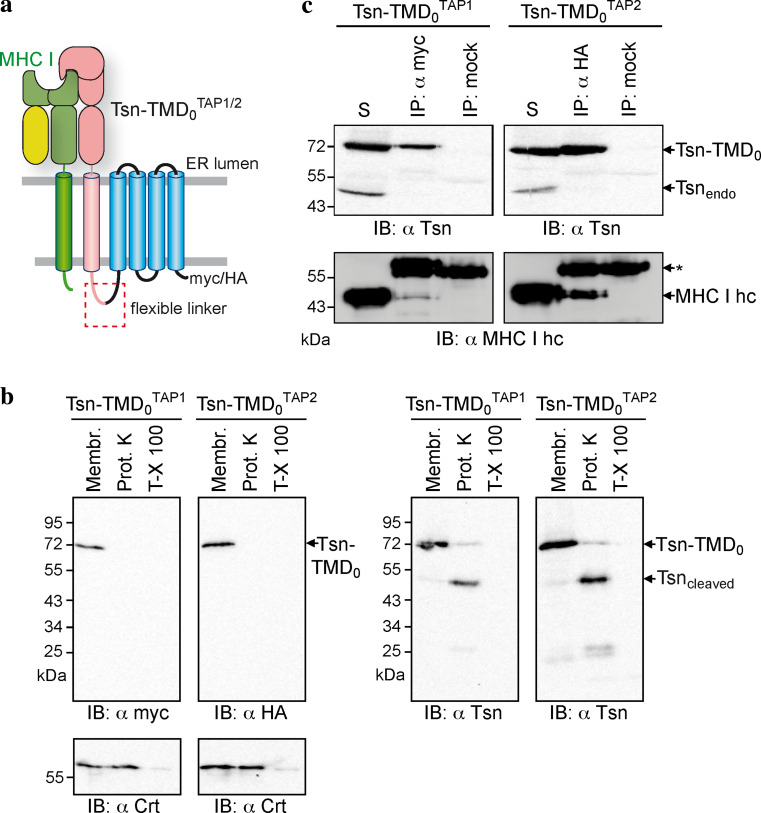
Each TMD_0_ harbors one single tapasin-binding site. **a** Model of the Tsn-TMD_0_^TAP1/2^ fusion constructs interacting with MHC I molecules. **b** Membrane topology of the Tsn-TMD_0_^TAP1/2^ fusion constructs. Crude membranes were prepared from HeLa cells transiently transfected with Tsn-TMD_0_^TAP1^ or Tsn-TMD_0_^TAP2^. Membranes (*Membr.*) were treated with Proteinase K (0.6 units) in the absence or presence of Triton-X 100 (*T*-*X 100*) for 30 min on ice followed by immunoblotting. **c** Tsn-TMD_0_^TAP1^ or Tsn-TMD_0_^TAP2^ were transiently expressed in HeLa cells and subjected to coimmunoprecipitation with anti-myc, anti-HA, or an isotype control antibody. *Asterisks*: immunoglobulin heavy chain. The solubilizate (*S*) represents 1/20 aliquot of the precipitate

How many tapasin molecules are present within a fully assembled PLC is still a matter of debate. The tapasin-to-TAP ratios reported varied from 1:1 and 2:1 to 4:1 [5, 27, 28]. To address this question, we tethered tapasin to the TMD_0_ of each TAP subunit via a flexible glycine–serine linker of 34 aa, leading to Tsn-TMD_0_^TAP1^ and Tsn-TMD_0_^TAP2^. As a result, the stoichiometry is fixed and one tapasin binding site is already occupied by the fused tapasin (Fig. 6a). Similar to the isolated TMD_0_s, the fusion constructs are located to the ER in HeLa cells, after transient expression (data not shown). Our recent experiments targeting the topology of TAP2 revealed that the N-terminus of TAP2 faces the cytosol. For further clarification, the membrane topology of the fusion constructs was confirmed by Proteinase K treatment (Fig. 6b). The myc- or HA-tag at C-terminus of Tsn-TMD_0_ was accessible from the cytosol, where the ER-lumenal calreticulin is degraded only after Triton-X 100 permeabilization of the membrane. In addition, the linker is accessible for Proteinase K, cleaving the tapasin from TMD_0_. These results demonstrate the correct membrane insertion of tapasin and that the linker and C-terminus of TMD_0_s are exposed to the cytosol. By coimmunoprecipitation, we could demonstrate that no additional, endogenous tapasin was recruited to Tsn-TMD_0_^TAP1^ or Tsn-TMD_0_^TAP2^ (Fig. 6c), revealing that there is only one single binding site for tapasin on each TMD_0_. Although Tsn-TMD_0_^TAP1^ recruited slightly less of MHC I than Tsn-TMD_0_^TAP2^, both Tsn-TMD_0_ fusion proteins were able to bind MHC I, showing that the fused tapasin is folded and functional.

## Discussion

In this study, we demonstrated that the isolated TMD_0_s of TAP1 and TAP2 are independently targeted to the ER membrane and form autonomous interaction hubs for tapasin recruitment. They are not required for the ER localization and transport function of the coreTAP complex [16]. A similar functional partition into a core ABC transport complex and extra N-terminal domains was shown for other ABC transporters. Some of these N-terminal extensions have an impact on the subcellular trafficking or function of the ABC transporter, while others seem to serve different purposes, extending the fascinating properties of ABC transporters towards receptor and channel functions. The lysosomal polypeptide transporter ABCB9 (TAP-like), which forms a homodimeric complex, possesses a TMD_0_ that can be expressed separately. Although this TMD_0_ is dispensable for mere transport activity, it is required for the lysosomal localization of the transporter. The core transport complex lacking the TMD_0_s is targeted to the plasma membrane but redirected into lysosomes upon separate expression of TMD_0_ [29]. However, an intrinsic affinity of TMD_0_ for the core translocation complex, as seen for ABCB9, was not observed for TAP (data not shown).

Mitochondrial ABC transporters, also members of the ABCB family, have N-terminal extensions containing very long mitochondrial pre-sequences [30–32]. In contrast to non-mitochondrial ABC transporters, these N-terminal extensions are not membrane spanning per se. Nevertheless, they are essential for correct targeting, since deletion of the leader sequence in ABCB10 and its yeast homologue MDL1 resulted in mistargeting of the transporter to the ER membrane [31, 32]. Vice versa, fusion of the N-terminal extension of ABCB7 (residue 1–135) to the dihydrofolate reductase leads to mitochondrial targeting of the fusion protein [30].

Most full-length ABC transporters of the subfamily C also display an extra N-terminal domain (named MSD or TMD_0_), which typically comprises five transmembrane-spanning segments (reviewed in [33]). MRP1 and MRP2, for example, harbor TMD_0_s with strategic functions in targeting of the transporters to the plasma membrane [34, 35]. As for TAP, the TMD_0_ of MRP1 is dispensable for transport activity per se [36]. The sulfonylurea receptor SUR1 (ABCC8) is the regulatory subunit of the K_ATP_ channel K_IR_6.2. Mutations in K_IR_6.2 are associated with congenital hyperinsulinism [37, 38] and neonatal diabetes [39, 40]. TMD_0_^SUR1^ is the interaction and regulation domain to K_IR_6.2 [41, 42]. The TMD_0_^SUR1^, expressed in the absence of the remaining core ABC transporter, retains its ability to associate with and gate K_IR_6.2 [43]. In conclusion, some ABC transporters present an interesting division of work using several independent membrane-spanning domains: one forming the gated translocation pathway and one with functions in regulation, trafficking, or binding of interaction partners. However, the TMD_0_s of TAP1 and TAP2 constitute prime examples of ER-resident ABC proteins.

A number of mechanisms are known that retain or retrieve proteins in or to the ER. Soluble proteins are retrieved from post-ER compartments by the KDEL motif that binds to the KDEL receptor. For type I membrane proteins, C-terminal di-lysine motifs (KKxx or KxKxx) interact with COPI vesicles for retrieval to the ER [44]. Type II membrane proteins are retained by a double-arginine motif at the N-terminus [45]. However, neither coreTAP nor any of the TMD_0_s contain these characteristic signals. Nevertheless, direct evidence for ER localization of both TMD_0_s is delivered in this work by immunofluorescence analysis. TMD_0_^TAP1^ and TMD_0_^TAP2^ were predominantly found in the ER, with a minor fraction entering the post-ER compartment, ERGIC. This distribution resembles that of wt TAP and is in agreement with a previous study that found a minor fraction of TAP active in the ERGIC [24]. Of note, the escape of TMD_0_^TAP2^ into the ERGIC is enhanced compared to TMD_0_^TAP1^. These data imply that the TMD_0_s of both TAP subunits are retained in the ER, but the unidentified retention or retrieval factor acts more strongly in TMD_0_^TAP1^ than in TMD_0_^TAP2^. This retention/retrieval signal must be directly located within each TMD_0_, because ER localization is not mediated by the interaction with the KKxx motif-containing partner tapasin. The localization of wt TAP was formerly suggested to be independent of tapasin [24]. Here, we demonstrate that this is also the case for the isolated membrane interaction hubs TMD_0_ of TAP1 and TAP2. In addition, we provide direct evidence that coreTAP1 and coreTAP2 localize to the ER membrane. Escape to post-ER compartments was weak and comparable to that of full-length TAP, demonstrating that the mechanism for TAP ER retention is preserved in coreTAP.

The observation that the independently expressed TMD_0_s of both TAP subunits are able to recruit tapasin gives direct evidence that the TMD_0_s are folded and functional independent of coreTAP. Furthermore, coimmunoprecipitation experiments with Tsn-TMD_0_ fusions demonstrate that each TMD_0_ only bears one single binding site for tapasin. This suggests a tapasin-to-TAP subunit ratio of 1:1, which is in perfect agreement with the latest study on this subject [28]. However, we cannot formally rule out the possibility that a second tapasin-binding site is formed by combination of the very C-terminal parts of TMD_0_^TAP1^ and TMD_0_^TAP2^ as well as the N-terminus of coreTAP1 and coreTAP2. Tsn-TMD_0_^TAP1^ was reproducibly found to recruit less MHC I than Tsn-TMD_0_^TAP2^ (Fig. 6). This might reflect a greater importance for TMD_0_^TAP2^ in MHC I loading. This is a reasonable speculation, since for chicken and all other avian MHC I loci sequenced so far, only TAP2 harbors a TMD_0_, while TAP1 has no TMD_0_ and is therefore equivalent to coreTAP1 [46, 47]. The exact interaction sites of TMD_0_^TAP1^ and TMD_0_^TAP2^ for tapasin remain unknown. For tapasin, a number of candidate residues have been proposed, including residues F397, F401, G405, K408, and W412 of mouse tapasin [48], and K408 of human tapasin [49]. For TAP, it was previously shown that removal of the first transmembrane helix destroys the tapasin-TAP interaction [17]. Whether this is due to an altered overall folding of the truncated N-terminal domain or missing essential residues will require future approaches. The structural analysis of the TMD is one approach to resolve this important interaction hub.

In conclusion, we show for the first time that the TMD_0_s of both TAP subunits form autonomous interaction scaffolds for the assembly of the MHC I peptide-loading complex in the ER membrane. Each TMD_0_ connects ERp57, calreticulin, and peptide-receptive MHC I via a single tapasin molecule to the peptide supplier TAP. According to this, the PLC can be subdivided into three functional modules: (1) peptide binding and transport by the coreTAP complex, (2) peptide loading and editing by the Tsn-ERp57/MHC I subcomplex, and (3) the conjunction of these functions, accomplished by the interaction hubs TMD_0_^TAP1^ and TMD_0_^TAP2^.

## Abbreviations

Crt: Calreticulin
ER: Endoplasmic reticulum
ERGIC: ER-*Golgi* intermediate compartment
MHC I: Major histocompatibility complex class I
PLC: Peptide-loading complex
TAP: Transporter associated with antigen processing
TMD: Transmembrane domain
Tsn: Tapasin
wt: Wild-type
